# Cryoablation for a right atrial myxoma arising from the Koch’s triangle: a case report

**DOI:** 10.1186/1749-8090-8-222

**Published:** 2013-12-02

**Authors:** Sotirios Marinakis, Dimiter Mircev, Pierre Wauthy

**Affiliations:** 1 Departement of Cardiac Surgery, Brugmann Hospital, Brussels, Belgium, Place Van Gehuchten 4, 1020 Brussels, Belgium; 2 Departement of Cardiology, Iris Sud Hospitals, Brussels, Belgium, Rue Jean Paquot 63, 1050 Brussels, Belgium

**Keywords:** Myxoma, Cardiac tumors, Pulmonary embolism, Heart failure

## Abstract

A 78-year-old caucasian patient with compromised cardiac function presenting recurrent episodes of pulmonary embolism was referred to our center for resection of a voluminous right atrial myxoma arising from the Koch’s triangle. To preserve the conduction system, we performed an excision of the myxoma associated with cryoablation of its stalk. This case is of special interest for discussing possibilities of preservation of the atrioventricular conduction system in such situations, provided that the contemporary literature does not propose concrete guidelines.

## Background

Myxomas are the most common primary heart tumors, accounting for 50% of all benign cardiac tumors. They predominate in women with a peak incidence in the third and sixth decades of life. About 75–80% of them arise from the left atrium and 15–20% from the right. They are pedunculated, and their stalk usually rises from the atrial septum [1,2].

## Case presentation

We report the case of a 78-year-old caucasian woman with a previous medical history of multiple pulmonary embolism who was referred to our cardiac surgery department with a diagnosis of a 5.6 × 3.6 cm right atrial myxoma after a second episode of pulmonary embolism. In 2004, after the first pulmonary embolism, a right atrial mass compatible with an intracardiac tumor or thrombus was detected, without further follow-up apart from an anticoagulation therapy stopped in 2007 because of a mild digestive hemorrhage.

**Figure 1:**
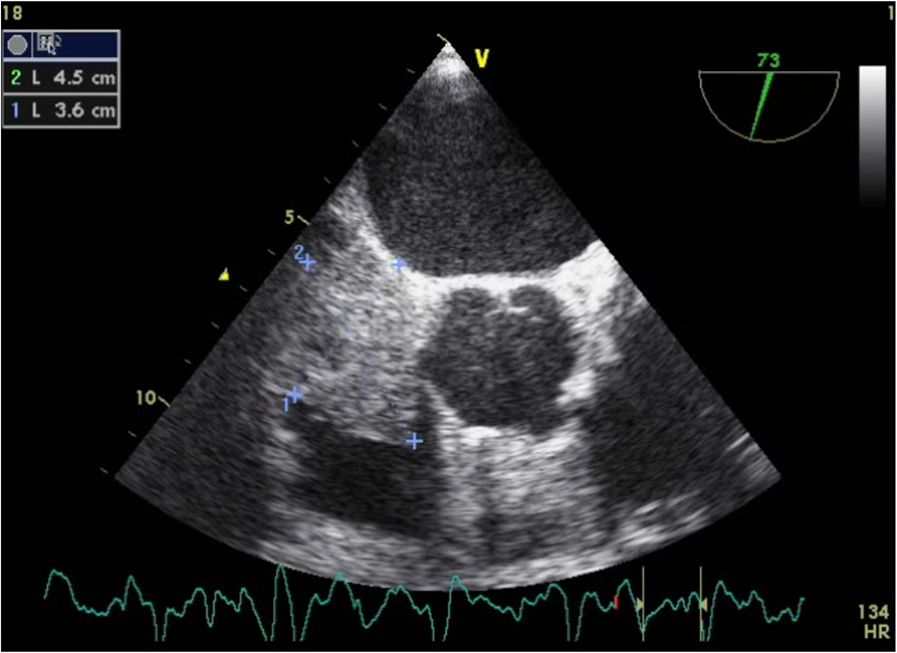
Transesophageal echocardiography demonstrating a myxoma of 5.6 × 3.6 cm rising from the right atrium.

The patient was admitted to a peripheral hospital for respiratory discomfort associated with right hemithorax pain radiating to the back. The electrocardiogram showed sinus rhythm, left ventricular hypertrophy, and 1 mm ST elevation in precordial leads compatible with a left ventricular hypertrophy. The laboratory analysis demonstrated mild acute renal failure, altered hepatic enzymes, and increased B-type natriuretic peptide (10,380 pg/mL). The pulmonary ventilation perfusion scan confirmed a mild pulmonary embolism. Transesophageal echocardiography (TEE) demonstrated a voluminous mass of 5.6 × 3.6 cm in the right atrium (Figure 1), protruding into the right ventricle, with its stalk fixed in the interatrial septum, findings that are echographically compatible with a myxoma. Furthermore, a dilatation of the left ventricle was observed, with diffuse hypokinesia and anteroseptal akinesia. The left ventricular ejection fraction (LVEF) was estimated to be 20%. At patient admission to our hospital, the coronarography demonstrated two significant stenoses of the middle (76–95%) and distal (76–95%) left anterior descending (LAD) artery and a non-significant (<50%) stenosis of the left main, circumflex, and right coronary arteries.

**Figure 2:**
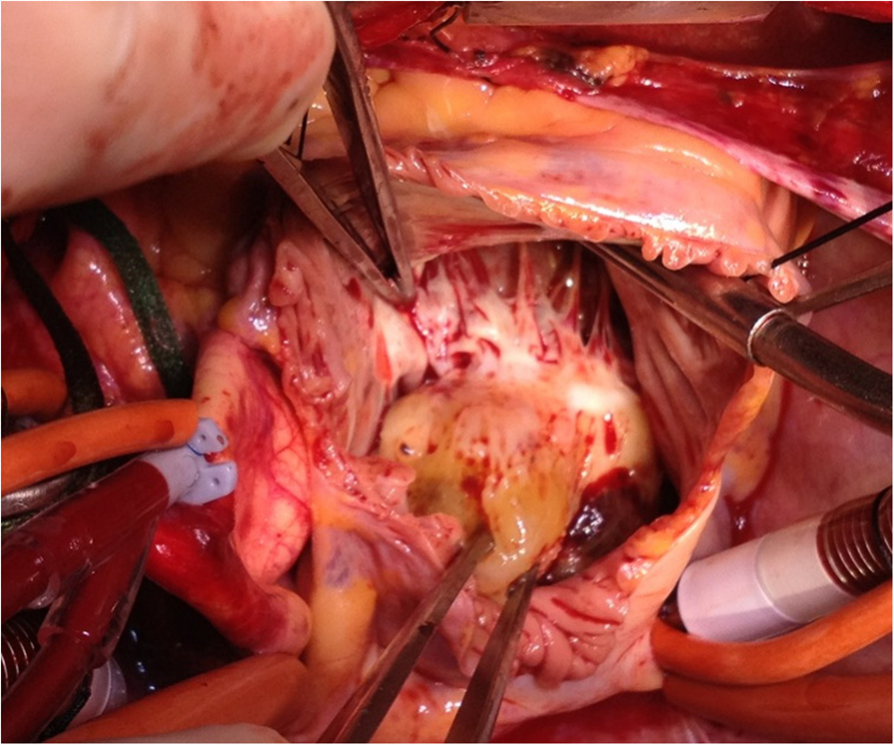
Per-operative vision of the myxoma in close relationship with the tricuspid valve.

The patient was urgently prepared for an excision of the myxoma and a coronary artery bypass grafting of the LAD artery with the left internal mammary artery (LIMA). The operation was performed through median sternotomy under cardiopulmonary bypass with bicaval cannulation and antegrade cold blood cardioplegia. After the coronary artery bypass graft anastomosis, a right atriotomy was performed. Following meticulous dissection of the myxoma, which was in close association with the anterior leaflet of the tricuspid valve, we noted that its stalk originated from the Koch’s triangle (Figure 2).

**Figure 3:**
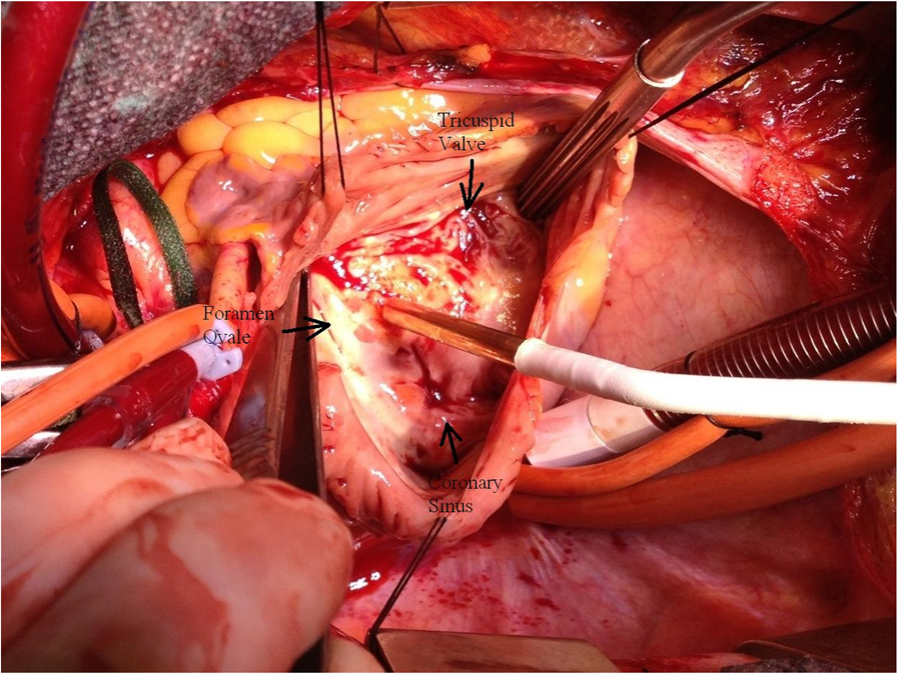
Cryoablation of myxoma’s stalk rising from the Koch’s triangle.

**Figure 4:**
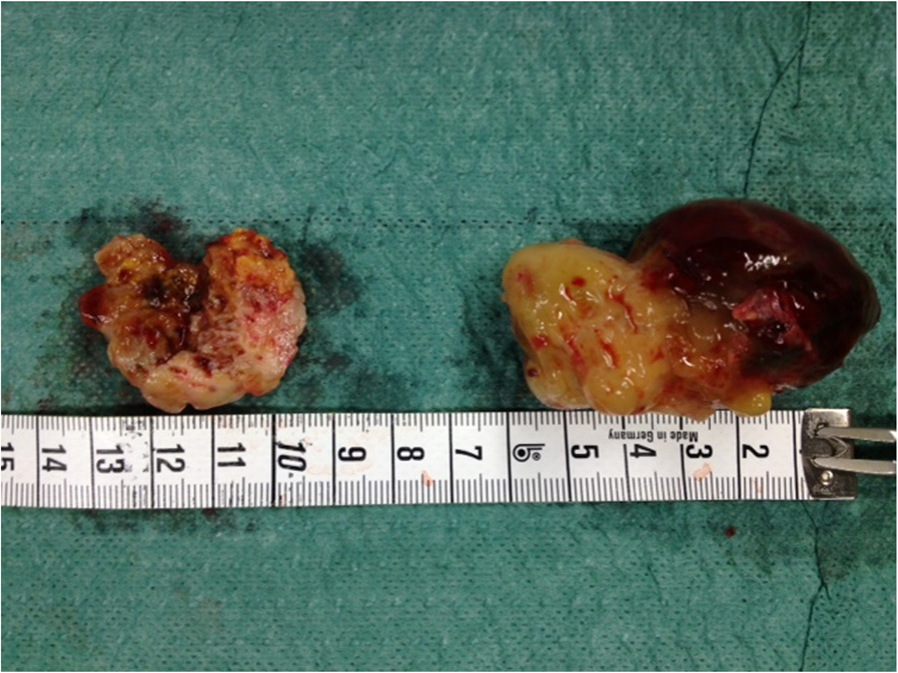
Pathologoanatomic specimen of the myxoma.

Given the fact that complete ablation with creation of an atrial septal defect in this area would result in a total atrioventricular block in a patient with major alteration of the LVEF, we decided to perform an excision of the myxoma at its base and a cryoablation (-110°C) of the insertion of its stalk for 60 seconds (Figure 3). The intraoperative TEE revealed no residual tumor, and the histopathologic examination of the specimen confirmed the diagnosis of a benign myxoma (Figure 4). The postoperative period was uneventful, and the patient presented no conduction problems in the perioperative and postoperative periods.

## Conclusions

Cryoablation of cardiac myxomas has been previously reported in a patient presenting with recurrent myxomas with good short-term results [3], but it has never been used, to our knowledge, as an alternative to atrial wall excision for myxomas rising from the Koch’s triangle. On the grounds of the severely compromised cardiac function and the high probability of a complete atrioventricular block associated with a classical excision of the full thickness of the adjacent atrial wall, we decided to perform only an excision of the root of the myxoma pedicle with cryoablation of its stalk. This case is of special interest because it describes an alternative excision strategy for myxomas situated near the conduction system. Limited excision of the the myxome without resection of its stalk is associated with a prohibitive risk of early recurrence [4,5]. As an alternative to a full thickness excision of the adjacent atrial wall to remove the myxome stalk which would necessitate a relatively extensive reconstruction to reach healthy tissue, we opted for a selective cryoablation of the stalk. In spite of the inherit risk of cryoablation to provoke conduction problems, this treatment remains less extensive than surgical excision and, therefore, of less risk to provoke a complete atrioventricular block. Certainly, the exact risk of tumor recurrence and conduction complications post cryoblation for treatment of myxomas rising close to conduction system needs further investigation.

Although in cardiac surgery there is little experience of cryoablation techniques concerning their oncologic result, previous reports from hepatic and other solid organ surgery support their efficacy [6-8]. Therefore, in difficult cases with critical cardiac function (particularly in older patients) and myxomas arising in precarious positions, cryoablation might be a reasonable alternative to radical surgical resection.

## Consent

Written information consent was obtained from the patient for publication of this Case report and any accompanying images. A copy of the written consent is available for review by the Editor-in-Chief of this journal.

## Abbreviations

TEE: Transesophageal echocardiography; LVEF: Left ventricular ejection fraction; LAD: Left anterior descending; LIMA: Left Internal mammary artery; C: Celsius.

## Competing interests

The authors declare that they have no competing interests.

## Authors’ contributions

The corresponding author Dr. SM had been involved in drafting the manuscript and collecting the data. He had, also, a substantial contribution to the concept and the design of the paper and in the interpretation of data. Dr. DM had been involved in revising critically the manuscript, for important intellectual content and he had given his final approval of the version to be published. Pr. Pierre Wauthy had a substantial contribution to the concept and the design of the paper and in the interpretation of data. He had, also, been involved in revising critically the manuscript, for important intellectual content and he had given his final approval of the version to be published. All authors read and approved the final manuscript.
